# Supplementary material for: Exploring nonlinear dynamics in periodically driven time crystal from synchronization to chaotic motion
Source: Nat Commun. 2025 Mar 26;16:2936. doi: 10.1038/s41467-025-58400-6 (PMC11937429; doi:10.1038/s41467-025-58400-6)
Supplement: Supplementary file 1 — Supplementary Information [file 41467_2025_58400_MOESM1_ESM.pdf]

Supplementary information for "Exploring nonlinear dynamics in periodically driven time crystal from synchronization to chaotic motion"

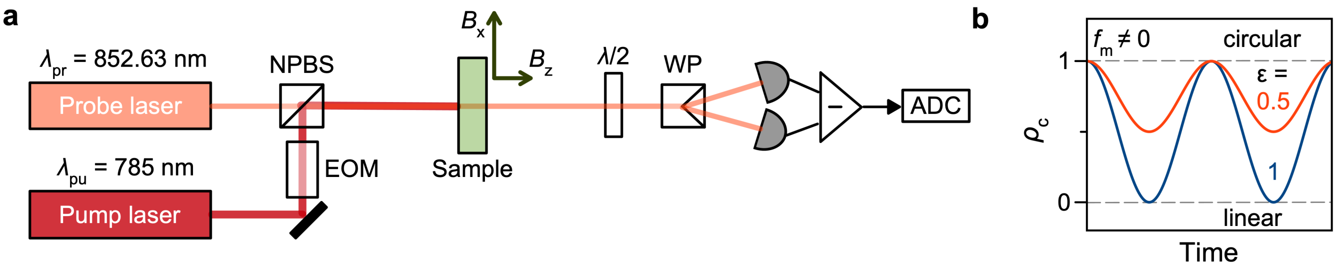

Supplementary Figure 1. **Implementation of the experiment.** (**a**) Scheme of the experimental setup. EOM is an electro-optical modulator for controlling the pump polarization, NPBS is a non-polarizing beam splitter, and WP is a Wollaston prism. ADC is an analog-to-digital converter. (**b**) Illustration of the sinusoidal change of the degree of circular polarization (ρ_c) leading to the polarization modulation with frequency f_m and two examples for the modulation depth, namely $\varepsilon = 0.5$ and $\varepsilon = 1$.

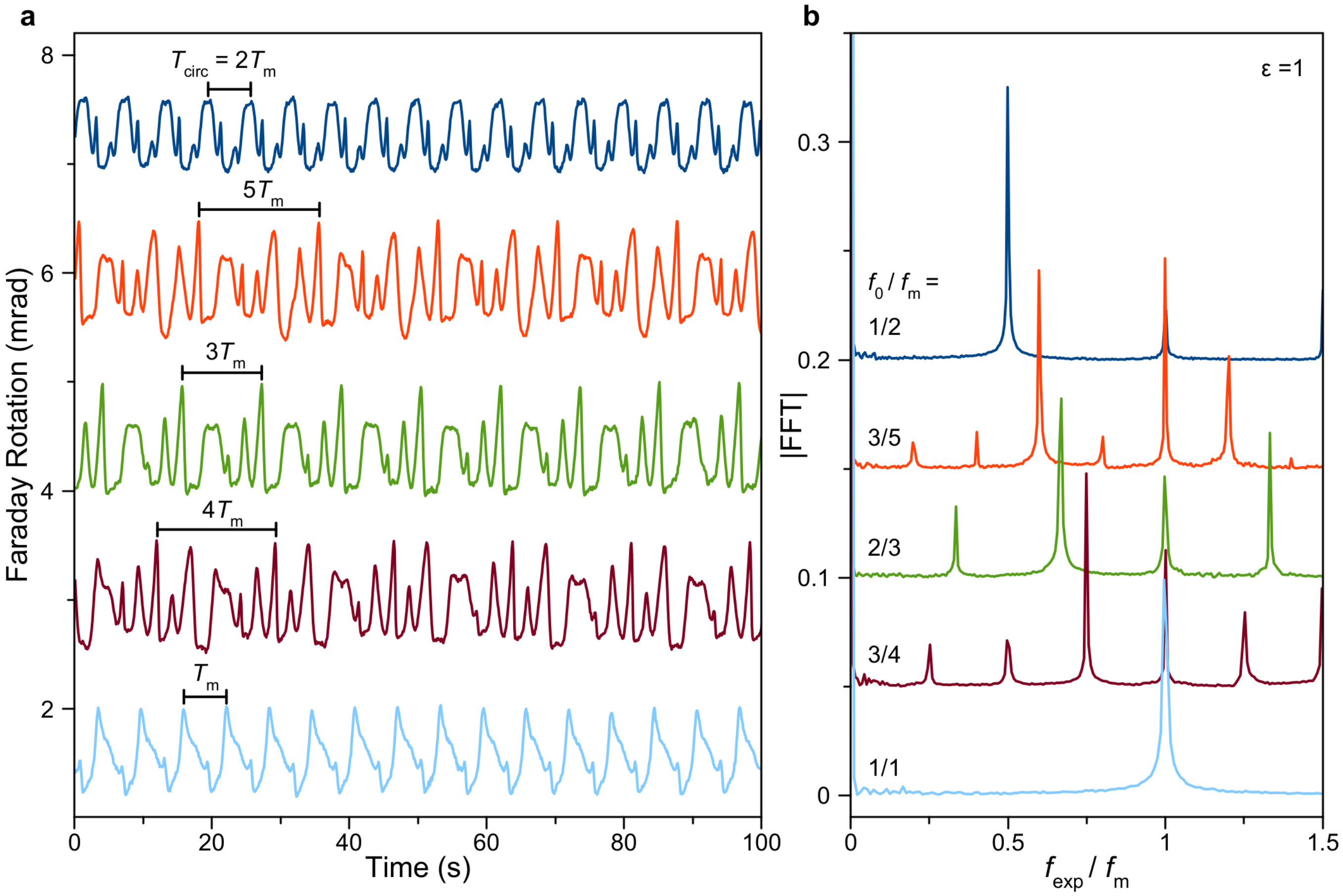

Supplementary Figure 2. **FR oscillations at different frequency fractions.** (**a**) Time traces measured at the entrainment plateaus for rising values of $f_0/f_m = 1/2, 3/5, 2/3, 3/4, 1/1$. The top blue-colored trace shows the $f_m = 2f_0$ case with the lowest frequency harmonic $f_{circ} = 1/T_{circ} = 1/2T_m$. The black bar shows the duration of the full periodicity interval T_{circ} in units of T_m. (**b**) Corresponding FFT spectra for 10-minute time traces as a function of the observation frequency f_{exp} normalized by f_m, $\varepsilon = 1$.

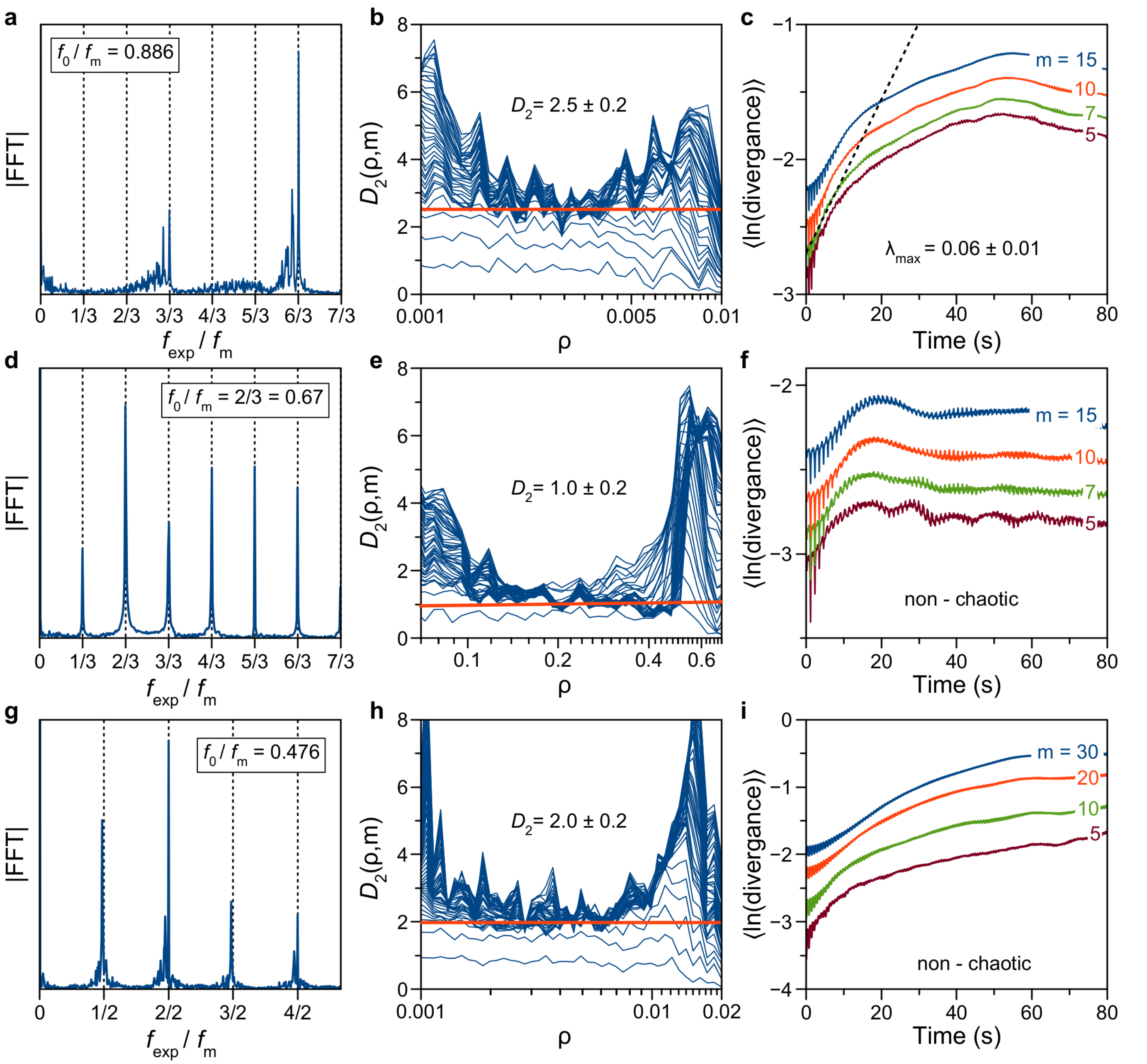

Supplementary Figure 3. **Nonlinear time series analysis.** (**a**) FFT spectrum at the edge of the entrainment plateau with $f_0/f_\mathrm{m} = 0.886$. (**b**) Slope of the correlation sums calculated for varying embedding dimensions ($m = 1 \div 30$) as a function of the threshold radius (ρ). The red line shows the linear range for $\rho = 0.001$ to 0.005 and gives the correlation dimension $D_2 = 2.5 \pm 0.2$, confirming the non-integer dimension. (**c**) Average logarithm of the divergence versus time for increasing embedding dimension. The maximal Lyapunov exponent is $\lambda_{\max} = 0.06$, confirming the divergence of chaotic trajectories in the phase space. (**d**) FFT spectrum at the center of the entrainment plateau with $f_0/f_\mathrm{m} = 2/3$. (**e**) The corresponding $D_2 = 1$ confirms the periodic limit cycle behavior. (**f**) The maximal Lyapunov exponent in this case is not positive. (**g**) FFT spectrum away from the edge of the entrainment plateau with $f_0/f_\mathrm{m} = 0.476$. (**h**) The corresponding $D_2 = 2$ confirms the quasi-periodic behavior. (**i**) The maximal Lyapunov exponent is not positive.

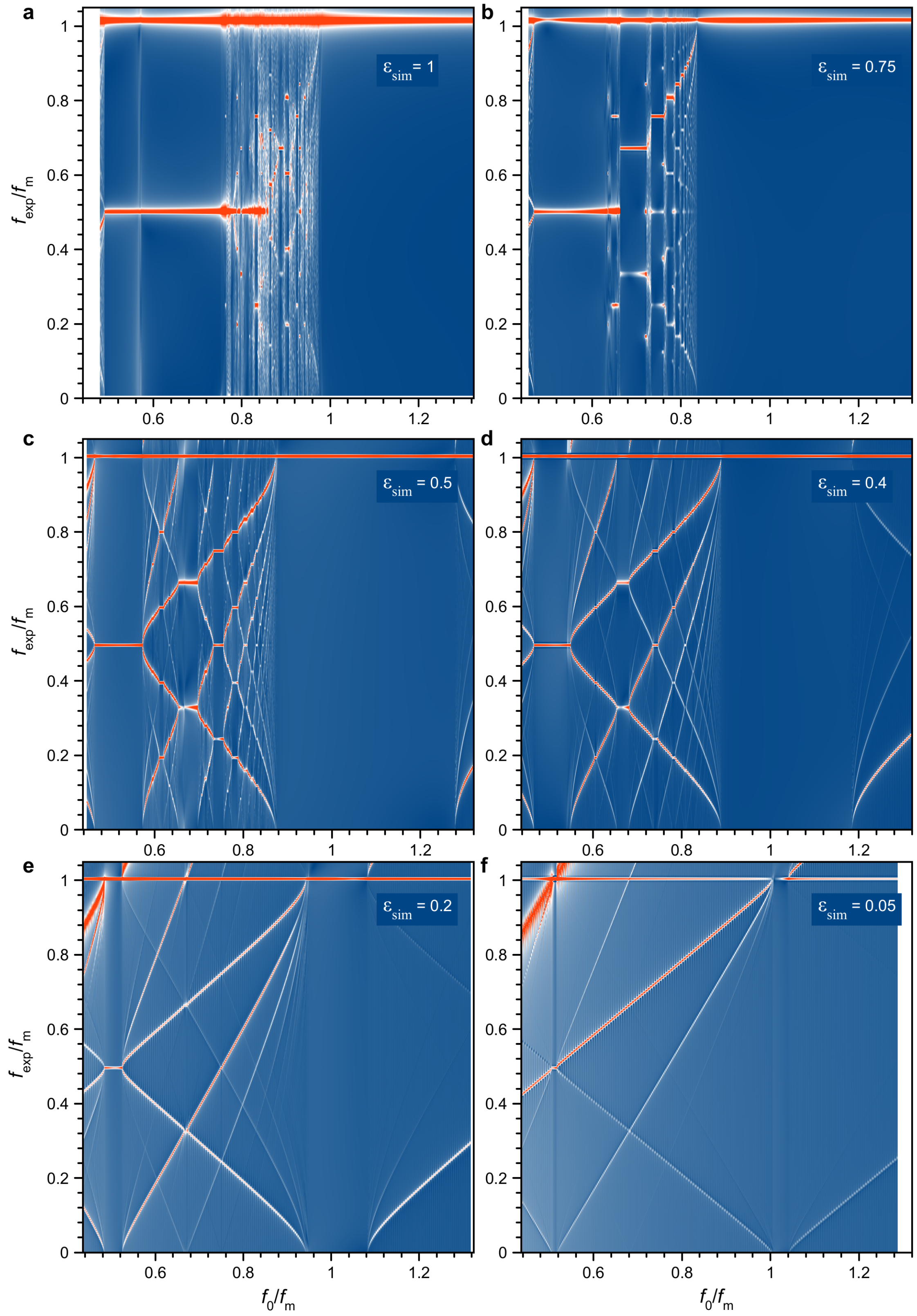

Supplementary Figure 4. **Periodically driven oscillations with varied modulation depth.** Contour plots of simulated FFT spectra as a function of the modulation rate $1/f_m$ normalized by the unperturbed auto-oscillation rate $1/f_0$ for varying values of ε_{sim}.

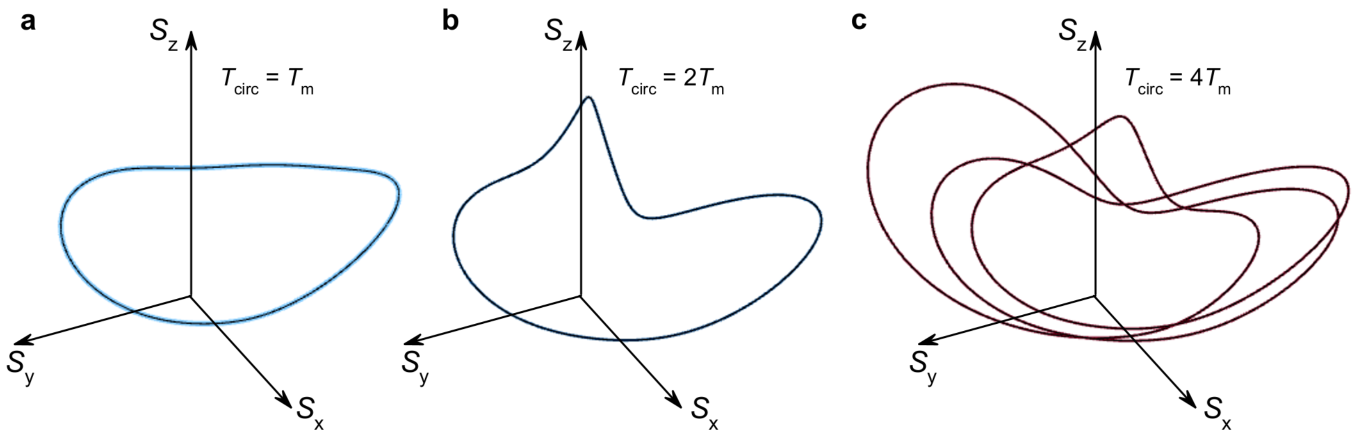

Supplementary Figure 5. **Simulated spin trajectories.** (**a**), (**b**), and (**c**), Simulated electron spin vector components evolution for $f_0/f_\mathrm{m} = 1/1$, $1/2$, and $3/4$, respectively. Each curve represents a limit cycle trajectory without intersection. The full cycle evolution time is the inverse of the effective winding number value of $T_\mathrm{circ}/T_\mathrm{m} = 1$, 2, and 4, respectively. $\varepsilon_\mathrm{sim} = 0.5$.

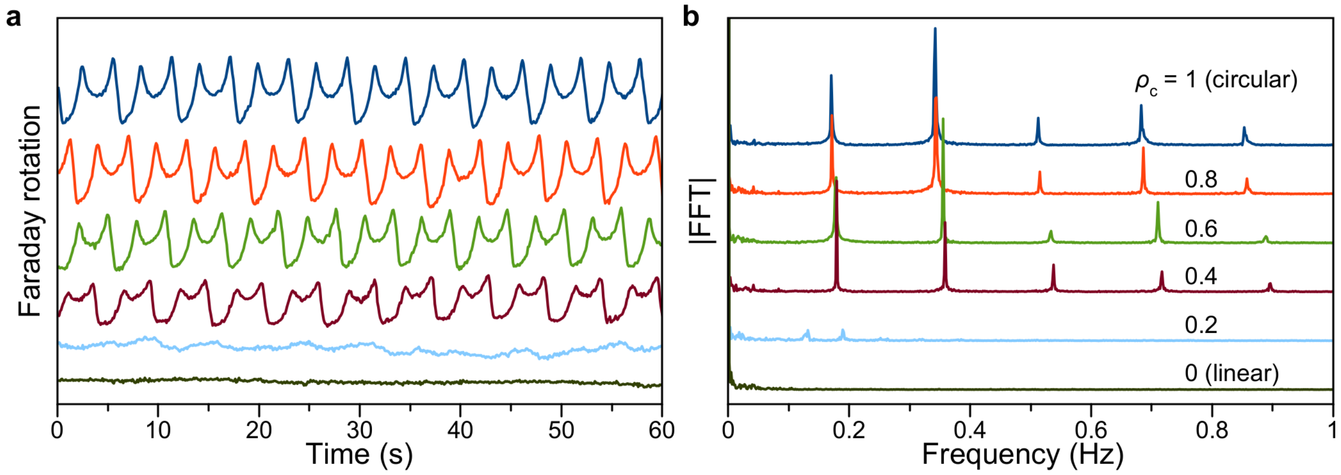

Supplementary Figure 6. **FR oscillations for different degrees of circular pump polarization.** (**a**) Time traces for different degrees of nonmodulated circular pump polarization. (**b**) Corresponding FFT spectra.

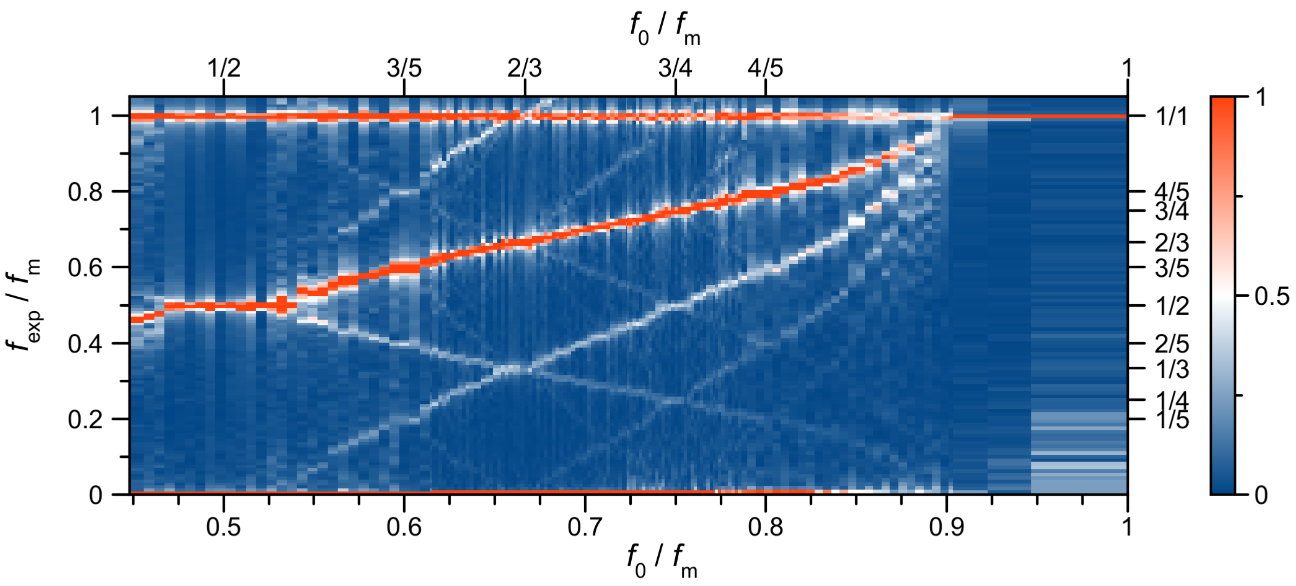

Supplementary Figure 7. **Entrainment plateaus and bifurcations.** Contour plot of experimental FFT spectra with the frequency axis $f_{\rm exp}$ normalized by the modulation frequency $f_{\rm m}$ as a function of the inverse modulation frequency, multiplied by the basic harmonic frequency. $\varepsilon = 1$. For each FFT, time traces of 10 minutes of recording time are used. The scale on the right shows the color scheme for the normalized amplitude of the contour maps.
